# DRUG REPURPOSING—Overcoming the translational hurdles to clinical use

**DOI:** 10.1002/prp2.548

**Published:** 2019-11-26

**Authors:** Jennifer H. Martin, Nikola A. Bowden

**Affiliations:** ^1^ Centre for Human Drug Research School of Medicine and Public Health University of Newcastle Hunter Medical Research Institute New Lambton Heights Australia

**Keywords:** area under the curve, cancer pharmacology, pharmacokinetics, repurposing

Drug repurposing is a methodology for identifying new uses for approved or investigational drugs outside the scope of the original medical indication. In the field of cancer drug development repurposing is of interest because despite the genomic revolution, therapeutic advances have been far slower than expected, possibly due to complicated regulatory and organizational challenges. However slow progress is also due to seemingly divergent research and political agendas supporting the focus on single intermittent and often non tumor representative genetic targets. This focus on “targets” has often ignored the well‐studied principles of cell biology, cancer biology and pharmacology.^1^ Similarly, since 2015, Obama and subsequent US Government's multibillion dollar investments into personalised medicine “delivering the right treatments, at the right time, every time to the right person” 2 whilst helpful to understand some aspects of disease pathophysiology, has also fueled single target focus. Among other things this methodology and research funding focus has also meant most drugs treating potential “targets” have not been found to be as useful as expected clinically, particularly in solid tumors.

Other issues contributing to the lesser gain than expected from the personalized medicine “revolution” include increasing financial targets for the pharmaceutical industry, with variable regulatory and reimbursement policies around the world, and the increasing costs for new drug development including multiple genetic subgroups and therefore larger studies required.

Importantly also from both a public health perspective and return on investment, the investments in personalized medicine projects may have come at the expense of other potential investments, occasioning concern in the greater health community particularly in nations with lower gross domestic products where important health funding trade‐offs need to be made.^3^

Lastly, with current drug development focus on “new” targets, there is a long lead time; it has become costly and the increasing regulatory requirement for larger studies with multiple subgroups is really quite inefficient. The ***repurposing strategy*** is therefore a potential solution because it offers various advantages over developing an entirely new drug, as dosing, toxicity, and efficacy data in different indications is often already known, as is knowledge on other issues affecting likelihood of drugs being used clinically, such as potential drug interactions, effects of food and other medicines. Further the regulatory and reimbursement costs are significantly reduced with repurposing as the need for early phase clinical trial work is less. For patients, the strategy provides more potential drug therapies including more combination therapy options. For clinicians, there are more options when toxicity and safety issues arise in specific populations, and there is often already knowledge around different pharmacology in different populations including vulnerable groups.

These thoughts and developments along the repurposing strategy are timely as new technologies are now available to help this aim and enable access to data and collaborations easier. There is interest also in streamlining aspects such as manufacturing, reformulation, regulatory and other organizational challenges and barriers. Further, many researchers have become interested in more systematic approaches to identify specific repurposable compounds for specific diseases. Over the last few years, a number of groups managing patients with few other therapeutic options particularly where there are a paucity of drugs and expensive medicine are looking at a variety of repurposing methods.^4^

The work of Tian et al in this Journal^5^ is therefore very timely. The team has taken an area of clinical need and has used a very cheap off patent drug that targets a key glycolytic pathway in cancer. Deregulated Krebs cycle and mitochondrial redox is a key requirement for cancer cells to divide faster than non cancer cells. Inhibiting the production of energy is therefore a key aim in cancer. As described in the article, dichloroacetate (DCA) can reverse the glycolytic process redirecting the metabolism of pyruvate away from lactate production and into mitochondrial oxidation by inhibiting of pyruvate dehydrogenase (PDH) kinases (PDKs).^6^, ^7^ Tian's team investigated the use of DCA for PDK inhibition in a small cohort of 7 multiple myeloma patients. *GSTZ1* genotypes in the protein coding and promoter regions, known to affect the rate and amount of enzyme activity were investigated in relation to DCA concentrations and peripheral neuropathy, the most common DCA associated toxicity.^8^

Overall, in this study, the −1002A/A homozygous GSTZ1 genotype was seen to be correlated with a 3‐fold increase in DCA trough concentrations, which in turn were correlated with both higher risks of neuropathy but better disease outcome. Interestingly, heterozygosity for −1002A was not associated with peak or trough DCA concentrations, indicating that homozygosity is required to see a phenotypic effect. Tian et al postulated that during DCA treatment individuals homozygous for −1002A will re‐express the GSTZ1 protein at a lower rate or amount, resulting in susceptibility to accumulation of DCA and increased peripheral neuropathy. The expected population frequency of 11% for the homozygous −1002A indicates a follow‐up analysis in the context of minimizing peripheral neuropathy when assessing alternative dosing approaches for DCA is warranted.

The pharmacogenomic effect of *GSTZ1* haplotypes is shown clearly in the two patients whose DCA half‐life of was shorter and AUC lower, correlating with heterozygosity for the high activity GSTZ1*A haplotype. This makes pharmacological sense, based on the mechanism of action of GSTZ1 in DCA metabolism, and the likely relationship of DCA exposure to activity. Although this was an interesting finding from the study, the difference due to this haplotype was not seen after 1 week of DCA treatment, indicating that the −1002A/A genotyping would be more beneficial for guiding safe use of DCA in future studies.

The follow‐up studies already underway are investigating alternate dosing regimens to achieve higher concentrations of DCA. The outcomes of these studies will be of great interest, as repurposing DCA for patients with high burden of disease in combination with standard treatments offers great hope for improving the outcomes for these patients.

Overall this study has achieved significant advances for the progression of new therapies for patients with stable disease, but with known certain future progression. Firstly it has shown the ability to do clinical studies with an old drug, for a new indication without extensive preclinical work up, using existing human pharmacokinetic data and standard pharmacological principles to deduce the starting dose. This work was undertaken in a hospital, in a real world clinical population, therefore having direct applicability to clinical practice and ability to be taken up.

As well as larger number of patients and longer follow‐up time of this cohort, the work would be enhanced for a clinical population if discussion around how the dose was selected was included, as well as why a loading dose was used. Long‐term, clinicians will want to know how long to give the drug for, in which combinations, when to do drug concentrations in the dosing cycle and how often to repeat it.

Lastly and importantly for a clinical setting, although Tian's conclusion focused on individualizing dosing regimens to achieve effective DCA concentrations while avoiding neuropathy, the ability of this therapeutic drug monitoring (TDM) service to occur in clinical practice is unclear. With other chemotherapy drugs, where dosing to Cmax, Cmin, or AUC has also been clearly shown to significantly benefit mortality,^9^, ^10^ uptake of this into practice has still been patchy. Genetic testing is another possibility, however the clearer correlation of exposure to outcome than genotype to outcome is seen in this study, as is known with other drugs such as azathioprine and fluoropyrimidines where both genotyping and phenotyping tests are available,^11^, ^12^ TDM could aid long‐term monitoring of dose, or even reduce the need for complex genotyping. It would also provide guidance as to whether the dose was too high or too low for that patient.

## DISCLOSURES

Nil.

## AUTHOR CONTRIBUTIONS

Equal.

## ETHIC STATEMENT

N/A
